# Green Design and Life Cycle Assessment of Novel Thiophene-Based Surfactants to Balance Their Synthesis Performance and Environmental Impact

**DOI:** 10.3390/ma18122701

**Published:** 2025-06-08

**Authors:** Catalina Stoica, Alina Roxana Banciu, Hisham Idriss, Justin Z. Lian, Anca-Maria Patrascu, Stefano Cucurachi, Sébastien Richeter, Sébastien Clément, Mihai Nita-Lazar

**Affiliations:** 1 National Research and Development Institute for Industrial Ecology—ECOIND, 57-73 Drumul Podu Dambovitei, District 6, 060652 Bucharest, Romania; catalina.stoica@incdecoind.ro (C.S.); alina.banciu@incdecoind.ro (A.R.B.); anca.harabagiu@ecoind.ro (A.-M.P.); 2 Institut Charles Gerhardt Montpellier (ICGM), University of Montpellier, Centre National de la Recherche Scientifique (CNRS) École Nationale Supérieure de Chimie de Montpellier (ENSCM), Balard Recherche, Pôle Chimie, ICGM UMR 5253, 1919 Rte de Mende, 34293 Montpellier Cedex 5, France; hisham.idrissk@gmail.com (H.I.); sebastien.richeter@umontpellier.fr (S.R.); sebastien.clement1@umontpellier.fr (S.C.); 3 Leiden University, Rapenburg 70, 2311 EZ Leiden, The Netherlands; z.lian@cml.leidenuniv.nl (J.Z.L.); s.cucurachi@cml.leidenuniv.nl (S.C.)

**Keywords:** polymeric thiophene-based surfactants, ecotoxicity, bacterial biodegradation

## Abstract

Continuous human population growth, industrialization, and technical progress have increased the demand for a new design and synthesis of chemical compounds. Developing eco-friendly chemical compounds has been a priority for fostering a sustainable and healthy environment, which is directly linked to human well-being. In this context, green chemistry and circular economy principles have been applied to generate valuable new chemicals, such as surfactants, with high market value. Surfactants play a crucial role in various products for both domestic and industrial applications, leading to their large-scale production a diverse array of chemical structures. However, the advantages of their use must be balanced against their negative environmental impact as pollutants. Thus, there is an increasing demand for the development of new eco-friendly surfactants. Additionally, life cycle assessment (LCA) studies of new surfactants are essential for evaluating their environmental impact, enhancing energy efficiency and facilitating the transition toward sustainable energy resources. In this work, we present the chemical synthesis of oligomeric and polymeric thiophene-based surfactants with potential applications in biosensors, organic transistors, and various other fields. The newly synthesized oligomeric and polymeric thiophene-based surfactants demonstrated medium-to-high biodegradation potential and showed no significant ecotoxicological effects on bacterial communities. However, the LCA of their synthesis revealed a negative impact on the environment and human health, particularly concerning polymeric thiophene-based surfactants. The LCA identified specific chemical steps that could be optimized to develop a new generation of eco-friendly surfactants.

## 1. Introduction

Surfactants or surface-active agents are chemical compounds that reduce surface tension by being adsorbed at surfaces and interfaces. They possess dual characteristics based on their hydrophilic and hydrophobic chemical structures and can be classified into four categories: anionic, cationic, nonionic, and zwitterionic [1]. Surfactants are involved in two main phenomena, adsorption and aggregation (e.g., micelles and other aggregated structures). Consequently, they are an important chemical source for both industrial and domestic applications. Surfactants are widely used as household cleaning and personal care products, as well as in various industrial applications including (i) emulsifiers in the food industry, (ii) additives in agriculture to enhance pesticides and herbicides to enhance adhesion to plant surfaces, (iii) oil recovery in the oil and gas industry, (iv) dyeing and printing in the textile industry, and (v) wastewater treatment [2,3,4].

Recent studies have focused on the synthesis of surfactants, particularly polymeric thiophene-based surfactants, which combined their electronic properties of conjugated thiophene backbones with their amphiphilic nature. These compounds have gained attention due to their potential applications in organic electronic [5,6,7,8], biological sensing and biomedical applications [9,10,11,12,13,14,15].

The economic success of using surfactants in various applications is counterbalanced by their environment presence as pollutants, especially in large urban areas [16]. There are three major methods for removing surfactants from wastewater, primarily employed by domestic and industrial wastewater treatment plants (WWTPs), physico-chemical methods (such as precipitation, adsorption, flocculation, electrolysis, sonication, oxidation with oxygen, ozone, H_2_O_2_/UV), and biological treatments (biodegradation).

Physico-chemical methods aimed at enhancing pollutant degradability can be quite costly. The reagents used for chemical oxidation of surfactants may produce final degraded products that are toxic than the original pollutant. Overall, these methods are often less appealing compared to biological treatments, which provide a low-cost and effective biotechnology for pollutant removal [17,18]. Biological treatments are conducted using anaerobic and aerobic bacterial communities from activated sludge in WWTPs, offering a low-cost and effective alternative for pollutant removal compared to the physical and chemical methods [18].

Nevertheless, surfactants are not effectively removed by WWTPs, as they can disrupt the biological treatment processes. Furthermore, biodegradation may produce more toxic byproducts than the parent compounds [19,20,21].

Unfortunately, cationic polythiophenes (e.g., quaternary ammonium derivatives) can break microbial membranes [22] thereby disrupting microbial communities essential for WWTP biological treatment or for maintaining environmental sustainability and biodiversity. Surfactants that reach freshwater systems may initially bioaccumulate in primary producers such as algae, subsequently affecting higher trophic levels [23,24]. Surfactants could induce alterations in some biological processes, such as oxidative stress parameters [25,26,27,28] or they may act as endocrine disruptors effect in vertebrates (fish) [28]. At the molecular level, various surfactants can bind to peptides, enzymes or DNA, causing abnormal folding of polypeptide chains or altering the surface charge of molecules, which can subsequently affect biological functions [29].

Currently, there is a significant interest in developing new surfactants, such as thiophene-based surfactants, which exhibit low environmental toxicity and high biodegradability. International norms/guidelines and requirements such as Regulation (EC) No. 648/2004 [30] have been established to ensure that detergents and surfactants can be marketed and used across the EU, while concurrently providing a high degree of protection for the environment and human health. Regulation (EC) No. 66/2010 [31] for EU Ecolabel set forth criteria for products with reduced environmental impact throughout their lifecycle, based on relevant scientific information, particularly regarding biodegradability and toxicity. Numerous biological methods exist to assess surfactants ecotoxicity, including tests on freshwater fish, aquatic and terrestrial plants, or benthic and planktonic crustaceans. However, European chemical safety guidelines have promoted the reduction of animal testing (especially on vertebrates) by introducing mandatory data-sharing requirements and encouraging the use of alternative testing methods such as using bacteria [32,33]. Biological models, especially bacteria cells, are particularly significant due to their robust adaptability to toxic compounds as well as their widespread presence in the environment. Bacteria have a simple structure and are therefore easy to use in evaluation studies, providing significant and rapid information regarding the potential toxic effects of chemical compounds. They enable rapid molecular-level detection of biological effects induced by specific chemical pollutants. Additionally, bacterial communities can respond to harmful pollutants by altering their population structures, making them non-specific environmental “sensors” for stress. Consequently, monitoring the harmful effects of chemicals or contaminated environmental samples on bacterial models has been an easier to use, more economical, and reproducible alternative to small organisms’ biological models such as invertebrates, algae, ciliates, rotifers, or plants [34]. In addition to the environmental impact of chemical compounds like oligomeric and polymeric surfactants, their entire chemical synthesis process, use, and disposal could be assessed by a life cycle assessment (LCA) methodology. LCA is a useful tool for evaluating the environmental impacts associated with all stages of a product’s life, from raw material extraction through production, use, and disposal [35,36]. It can help technology developers and industries identify opportunities for reducing environmental impacts and improving sustainability. In the field of chemical synthesis, LCA allows researchers and manufacturers to evaluate the environmental impacts of various chemical processes and reactions, considering factors such as carbon footprint, energy consumption, emissions, and resource depletion at each stage of the synthesis [37,38]. Moreover, LCA empowers chemists to compare different synthesis pathways and select the most sustainable options, crucial for developing greener chemical processes, which optimize resource use and minimize waste [38,39].

In this work, we analyzed the chemical synthesis process of new thiophene-based surfactants with a wide range of potential applications in electron transfer for biomimetic devices and sensors based on LCA criteria. The LCA analysis of the entire chemical synthesis process demonstrated that 3,3″-didodecyl-3′-(2-(2-(2-methoxyethoxy)ethoxy)ethoxy)methylterthiophene (3DT-3TEGT) was less toxic to humans and the environment compared to poly(3-hexylthiophene)-*block*-poly(3′-(2-(2-(2-methoxyethoxy)ethoxy)ethoxy)methylthiophene) (P3HT-*block*-P3TEGT) and poly(3-hexylthiophene)-*ran*-poly(3′-(2-(2-(2-methoxyethoxy)ethoxy)ethoxy)methylthiophene) (P3HT-*ran*-P3TEGT). The biodegradability and ecotoxicological tests revealed a reversed hierarchy, where P3HT-*block*-P3TEGT and P3HT-*ran*-P3TEGT surfactants exhibited greater biodegradability and lower toxicity towards the biological models tested than 3DT-3TEGT.

The oligomeric and polymeric surfactants derived from thiophene compounds exhibited significant biodegradability potential in the presence of microbial communities isolated from a fully functional municipal WWTP. Their biodegradability potential aligns well with the ecofriendly properties, making biomimetic devices and sensors based on these chemical compounds likely to have a very low environmental impact.

## 2. Materials and Methods

### 2.1. Materials

Chemical reactions based on Schlenk techniques were performed in an inert atmosphere under argon gas. All anhydrous solvents and reagents were obtained from commercial suppliers: 2-bromo-3-hexyl-5-iodothiophene (Fluorochem, 95%), isopropyl magnesium chloride solution (Merck, Darmstadt, Germay), and [1,3-bis(diphenylphosphine)propane]-nickel(II) dichloride (NiCl_2_(dppp) (TCI, 98%).

Tetrakis(triphenylphosphine)palladium (0) catalyst, 3-dodecyl-2-(4,4,5,5-tetramethyl-1,3,2-dioxaborolan-2-yl)thiophene (1), and 2,5-dibromo-3-(2-(2-(2-methoxyethoxy)ethoxy)ethoxy)methylthiophene (2) were prepared according to Coulson et al., 1972 [40], Kong et al., 2009 [41], and Lee et al., 2011 [42]. TLCs were carried out on Merck DC Kieselgel 60 F-254 aluminum sheets and spots were visualized with a UV-lamp (λ = 254/365 nm) if necessary. Preparative purifications were performed by silica gel column chromatography (Merck 40−60 μM) and flash chromatography was carried out using Biotage^®^ Isolera™ Systems (Biotage, Uppsala, Sweden) (UV-Vis 200 nm–800 nm detector) over silica cartridges (Sfär HC D).

### 2.2. Characterization Methods

Nuclear magnetic resonance (NMR) spectroscopy and mass spectrometry (MS) were performed at the Laboratoire de Mesures Physiques (LMP) of the University of Montpellier (UM). The 1H and 13C{1H} NMR spectra were recorded on Bruker 400 MHz Avance III HD and 500 MHz Avance III spectrometers at 298 K. CDCl3 (99.8%) (Sigma-Aldrich, Saint Louis, MO, USA) was used as received. The 1H and 13C{1H} NMR spectra were calibrated using the relative chemical shift of the residual non-deuterated solvent as an internal standard. Chemical shifts (δ) were expressed in ppm. Abbreviations used for NMR spectra were as follows: s, singlet; d, doublet; t, triplet; m, multiplet.

High-resolution mass spectra (HRMS) were recorded on a Bruker MicroTof QII instrument in positive/negative modes (ESI). Number-averaged (M_n_) and weight-averaged (M_w_) molecular weights and the molecular weight distribution (Ð) of P3HT-*block*-P3TEGT and P3HT-*ran*-P3TEGT were measured using size exclusion chromatography (SEC) on a Polymer Laboratories liquid chromatograph equipped with a PL-DG802 degasser, an isocratic HPLC pump LC 1120 (flow rate of 1 mL min^−1^), a Marathon autosampler (loop volume of 200 mL, solution concentration of 1 mg mL^−1^), a PL-DRI refractive index detector, and three columns: a PL gel 10 mm guard column and two PL gel Mixed-B 10 mm columns (linear columns for the separation of molecular weight polystyrene standards ranging from 500 to 10^6^ Da). The eluent used was tetrahydrofuran (THF) at a flow rate of 1 mL.min^−1^ at 35 °C. Polystyrene standards were used to calibrate the SEC.

### 2.3. Synthesis of Oligomeric and Polymeric Surfactants

The 3DT-3TEGT synthesis was based on methods described by Idriss et al. (2025) [43]. Briefly, 3-dodecyl-2-(4,4,5,5-tetramethyl-1,3,2-dioxaborolan-2-yl)thiophene (**1**) (1.066 mmol) and 2,5-dibromo-3-(2-(2-(2-methoxyethoxy)ethoxy)ethoxy)methylthiophene (**2**) (0.216 g, 0.520 mmol) were placed under argon into a two-necked 100 mL round-bottom flask with anhydrous THF (12 mL). Next, 4 mL of a 2 M sodium carbonate aqueous solution was then added and the mixture was stirred at room temperature for 10 min. After the addition of tetrakis(triphenylphosphine)palladium(0) catalyst (0.018 g, 0.016 mmol), the mixture was refluxed for 24 h. After cooling to room temperature, the reaction was quenched with water (20 mL). The organic phase was then extracted with dichloromethane (3 × 50 mL), dried over anhydrous MgSO_4_, filtered, and the solvent was removed under reduced pressure. The crude product was then purified by column chromatography on silica gel using a mixture of hexane and ethyl acetate (7/3, *v*/*v*) as an eluent leading to 3DT-3TEGT as a yellow oil (0.21 g, 52%). ^1^H NMR (400 MHz, CDCl_3_) δ = 7.32 (d, 1H, ^3^*J*_H-H_ = 5.2 Hz), 7.18 (s, 1H), 7.15 (d, 1H, ^3^*J*_H-H_ = 5.2 Hz), 6.96 (d,1H, ^3^*J*_H-H_ = 5.2 Hz), 6.92 (d, 1H, ^3^*J*_H-H_ = 5.2 Hz), 4.42 (s, 2H), 3.64-3.36 (m, 18H), 2.77 (t, 2H, ^3^*J*_H-H_ = 7.8 Hz), 2.55 (t, 2H, ^3^*J*_H-H_ = 7.8 Hz), 1.63–1.24 (m, 41H), 0.85 (m, 6H). ^13^C{^1^H} NMR (126 MHz, CDCl_3_) δ 142.9, 139.8, 138.4, 136.7, 131.6, 130.5, 130.2, 129.0, 127.4, 127.1, 125.9, 123.8, 72.0, 70.8, 70.70, 69.5, 66.9, 59.2, 32.0, 31.0, 30.8, 29.8, 29.7, 29.6, 29.5, 29.0, 22.8, 14.2. HRMS (ESI+) *m*/*z* calcd for C_44_H_76_NO_4_S_3_ [M + NH_4_]^+^ 778.4931 Da, found: 778.4932 Da.

P3HT-*block*-P3TEGT. In two two-neck round-bottom flasks (100 mL) equipped with a magnetic bar, 2-bromo-3-hexyl-5-iodothiophene (**3**) (0.558 g, 1.50 mmol), and **2** (0.21 g, 0.5 mmol) were dried under vacuum after heating at 50 °C (magnetic heating stirrer) during 10 min, and then cooled at room temperature. Anhydrous THF (10 mL) was then added for each flask by using a cannula, and then the solutions were stirred at 0 °C for 15 min. Isopropylmagnesium chloride (2.0 M in THF) (0.75 mL and 0.25 mL) was then transferred to, respectively, **3-** and **2**-containing flasks using a syringe after which the mixtures were stirred at 0 °C for 1 h. NiCl_2_(dppp) (18 mg, 0.03 mmol) was then added to the solution containing **2** after warming the solution at room temperature. The solution containing **2** and the catalyst was then stirred for 2 h before the addition of solution containing **3**. The resulting mixture was then left under stirring overnight. The reaction was finally quenched by the addition of MeOH followed by precipitation in MeOH (200 mL) and filtration. The resulting polymer was then purified by Soxhlet extraction in methanol/acetone/hexane and chloroform (24 h each time, 300 mL solvent, balloon heater). The chloroform solution was then concentrated (from 300 to 30 mL) and methanol (300 mL) was added to precipitate the polymer. The polymer was isolated by filtration (0.20 g, 54%). SEC (THF, PS standards): M_w_ = 3500 g mol^−1^, M_n_ = 3000 g mol^−1^, Đ = 1.15. ^1^H NMR (400 MHz, CDCl_3_) δ = 6.98 (s, ArH, 1H), 4.68 (s, -CH_2_-O), 3.78–3.48 (m, -CH_2_-O), 3.35 (s, -OCH_3_ (P3TEGT)), 2.82–2.39 (m, -CH_2_ (P3HT)), 1.72–1.28 (m, -CH_2_-), 0.91 (s, -CH_3_ (P3HT), 3H) ppm.

P3HT-*ran*-P3TEGT. In two two-neck round-bottom flasks (100 mL) equipped with a magnetic bar, **3** (0.58 g, 1.55 mmol) and **2** (0.62 g, 1.48 mmol) were dried under vacuum after heating at 50 °C (magnetic heating stirrer) for 10 min, and then cooled at room temperature. Anhydrous THF (10 mL) was then added for each flask by using a cannula and then the solutions were stirred at 0 °C for 15 min. Isopropylmagnesium chloride (2.0 M in THF) (0.77 mL and 0.74 mL) was then transferred to, respectively, **3**- and **2**-containing flasks using a syringe after which the mixtures were stirred at 0 °C for 1 h. NiCl_2_(dppp) (48 mg, 0.09 mmol) was then added to the solution containing **3** followed by the solution containing **2**. The mixture was then stirred overnight. The reaction was finally quenched by the addition of MeOH followed by precipitation in MeOH (200 mL) and filtration. The resulting polymer was then purified by Soxhlet extraction in methanol/acetone/hexane and chloroform (24 h each time, 300 mL solvent, balloon heater). The chloroform solution was then concentrated (from 300 to 30 mL) and methanol (300 mL) was added to precipitate the polymer. The polymer was isolated by filtration (0.35 mg, 52%). SEC (THF, PS standards): M_w_ = 3900 g mol^−1^, M_n_ = 2600 g mol^−1^, Đ = 1.49. ^1^H NMR (400 MHz, CDCl_3_) δ = 6.99 (s, ArH, 1H), 4.64 (s, -CH_2_-O), 3.76-3.62 (m, -CH_2_-O),), 3.53 (m, -CH_2_-O),3.34 (s, -OCH_3_ (P3TEGT)), 2.78-2.36 (m, -CH_2_ (P3HT)), 1.69-1.28 (m, -CH_2_-), 0.91 (s, -CH_3_ (P3HT), 3H) ppm.

### 2.4. Dynamic Light Scattering (DLS)

Dynamic light scattering (DLS) measurements were performed using a Malvern Zetasizer Nano series Nano-ZS (Malvern Panalytical, Worcestershire, UK) in water. The critical micellar concentration (CMC) values were determined by preparing different solutions in water at different concentrations and measuring the intensity of the scattered light. The data were visualized by plotting scattered light intensity as a function of the concentration, revealing a sharp increase at the CMC. The underlying principle is that larger particles scatter light more efficiently than smaller molecules.

### 2.5. Biodegradation of Oligomeric and Polymeric Surfactants

The biodegradation potential of oligomeric and polymeric surfactants (3DT-3TEGT, P3HT-*block*-P3TEGT and P3HT-*ran*-P3TEGT) was analyzed based on the biochemical oxygen demand (BOD_5_) (SR EN ISO 5815-1:2020 method) [44] and chemical oxygen demand (COD) (SR ISO 15705:2022 method) [45] ratio, referred to as the Biodegradability Index (Table 1).

The BOD_5_/COD ratio of ≥0.5 indicates that a tested substance could be biologically oxidized and it is highly biodegradable in the environment. BOD_5_/COD ratio between 0.2 and 0.5 suggests that the tested substance has medium biodegradation potential in the environment, while a ratio of <0.2 indicates that the compounds are non-biodegradable [46].

Respiration tests were performed by incubating a bacterial consortium isolated from a municipal WWTP activated sludge in the presence of oligomeric and polymeric surfactants (3DT-3TEGT, P3HT-*block*-P3TEGT, and P3HT-*ran*-P3TEGT) according to SR EN ISO 8192:2008 standard method [47] (similar with the Organization for Economic Cooperation and Development (OECD) guideline no. 209). Briefly, a bacterial consortium was incubated in the presence of 100 mg/L oligomeric and polymeric surfactants at a constant temperature of 20 ± 2 °C up to 1 h. The oxygen consumption (respiration rate) of each test was measured with an oxygen-sensitive electrode system (Orion Star A329, Thermo Scientific, Bremen, Germany) every 15 min.

### 2.6. Bacterial Growth Inhibition Tests

Two non-pathogenic Gram-negative bacterial strains *Escherichia coli* (*E. coli*) (NCTC 12241) (National Collection of Type Cultures, Salisbury, UK) and *Pseudomonas putida* (*P. putida*) (ATCC 17514) (American Type Culture Collection, MD, USA) and one non-pathogenic Gram-positive bacteria strain *Lactobacillus acidophilus* (*L. acidophilus*) (ATCC 4356) (American Type Culture Collection, USA) were used in the study. Bacterial strains *L. acidophilus*, cultured on MRS broth (VWR Chemicals, Belgium), *E. coli*, and *P. putida*, cultured on Lauryl tryptose broth (Scharlau, Barcelona, Spain), were incubated in the absence or presence of oligomeric and polymeric surfactants (3DT-3TEGT, P3HT-*block*-P3TEGT, and P3HT-*ran*-P3TEGT) at 37 °C for 24 h under gentle rotation at 130 rpm (New Brunswick Scientific, Innova 44, Eppendorf, Hamburg, Germany). The effect of these compounds on bacterial growth was spectrophotometrically monitored at a wavelength of 600 nm (OD_600nm_), using a UV-VIS spectrometer (VWR International, PA, USA).

### 2.7. Non-Ionic Surfactant Detection

The concentration of nonionic surfactants was determined by UV spectrophotometric measurement. Briefly, surfactants were mixed with Dragendorff reagent and the formed bismuth-surfactants pellet was solubilized with ammonium tartrate. The concentration of bismuth, directly equivalent to the concentration of the surface agent, was optically measured at 263.5 nm on a spectrophotometer Shimadzu (Shimadzu, Kyoto, Japan). The calibration curve was made using Triton-X (Sigma-Aldrich, Saint Louis, MO, USA) as the certified material.

### 2.8. Life Cycle Assessment of Surfactants Synthesis Process

A life cycle assessment (LCA) was conducted to evaluate the environmental impacts of synthesizing 3DT-3TEGT, P3HT-*block*-P3TEGT, and P3HT-*ran*-P3TEGT. The system boundary for the LCA of the environmental impacts of the oligomeric and polymeric surfactant molecules (3DT-3TEGT, P3HT-*block*-P3TEGT, and P3HT-*ran*-P3TEGT) was defined as cradle-to-gate, with the functional unit set as 1 gram of surfactant synthesized in Europe.

**Figure 1:**
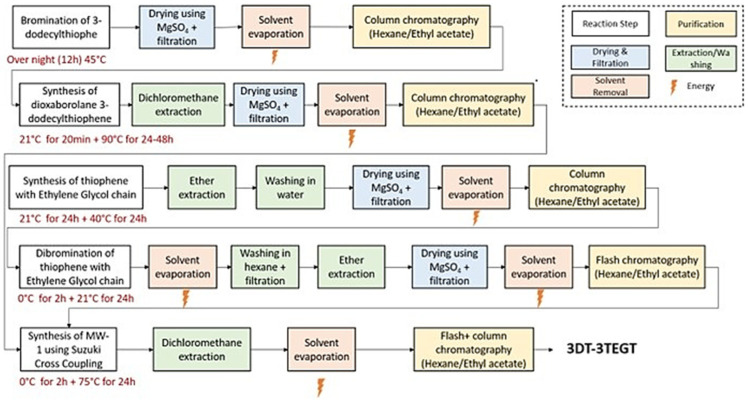
Onsite life cycle inventory data collection process based on 3DT-3TEGT chemical synthesis.

The LCA software used was Activity Browser V2.10.0, and the database utilized was ecoinvent 3.9, with the EF family method applied for the environmental impact assessment. Life cycle inventory (LCI) data were collected directly from the laboratory within the Department of Chemistry at the University of Montpellier and validated by experts here. A flowchart was created to depict the entire synthesis process for the targeted oligomeric and polymeric surfactants (Figure 1). The material consumption of various related chemicals and the energy consumption of many processes were recorded. The LCI data, along with detailed information, are provided in Tables S1 and S2 of the Supporting Information. Chemical stoichiometry methods were used to derive the target chemicals and calculate their environmental impacts, focusing on chemicals not included in the ecoinvent database but involved in the chemical synthesis process. A contribution analysis was conducted to identify key contributors and stages with high environmental impacts, followed by recommendations to improve the synthesis process based on the LCA results.

## 3. Results and Discussions

### 3.1. Synthesis of Thiophene-Based Surfactants

**Figure 2:**
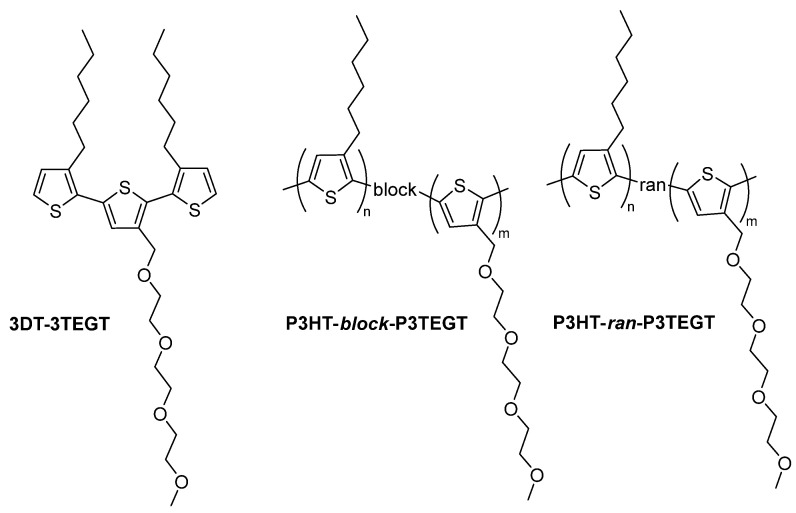
Structure of thiophene-based surfactants.

Series of amphiphilic thiophene-based oligomers and polymers (3DT-3TEGT vs. P3HT-*block*-P3TEGT and P3HT-*ran*-P3TEGT) were developed and linked in an α,ω (1,4)-pattern to maintain conjugation and preserve their optoelectronic properties (Figure 2).

The selection of the thiophene platform was driven by its status as a benchmark material for optoelectronic applications, owing to its tunable optical and electronic properties, which can be readily adjusted through well-established synthetic techniques [48]. The ability of amphiphilic oligothiophenes and polythiophenes to self-assemble in water into a wide range of structures such as spheres, elongated micelles, bilayer structures, core-shell discs or cylinders in solution, depending on the conjugation length, the architecture (block or graft copolymers), or the nature of the polar headgroup (cationic, nonionic ethylene oxide, peptide) [49,50,51,52,53,54,55,56]. Following this strategy, we developed a series of amphiphilic thiophene-based oligomers and polymers, which incorporate hydrophobic alkyl chains and nonionic ethylene glycol hydrophilic chains and with different conjugation length (number of thiophene units). The polar ethylene glycol group was selected for its better solubility in organic solvents compared to ionic groups, simplifying the purification process. Random and block copolymers were also prepared, allowing for the examination of the effect of conjugation length. Random copolymers are easier to synthesize than block or graft copolymers and allow for a broader selection of monomers [57,58]. Studies highlight their advantages, including cost-effectiveness and simpler characterization [59,60,61].

**Figure 3:**
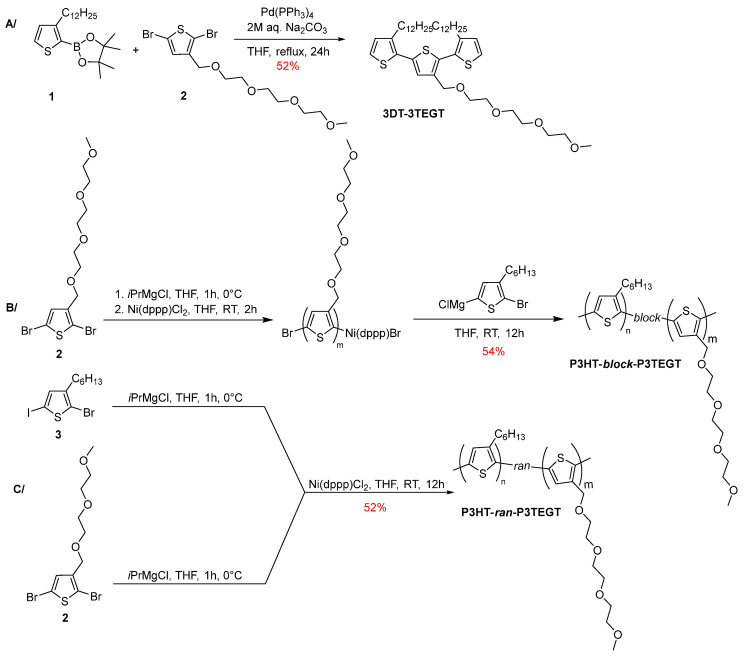
Synthetic pathway towards thiophene-based oligomeric and polymeric surfactants: (**A**) 3DT-3TEGT; (**B)** P3HT-*block*-P3TEGT; (**C**) P3HT-*ran*-P3TEGT.

3DT-3TEGT was prepared by a palladium-catalyzed Suzuki cross-coupling reaction of hydrophobic and hydrophilic thiophene building blocks (Figure 3A) and isolated in a 52% yield [43]. Its structure was confirmed by combining NMR spectroscopy and mass spectrometry (Figures S1–S3 in the Supporting Information). Notably, high-resolution ESI-TOF mass spectrometry (positive mode) confirmed the presence of pseudo-molecular ion [M + NH_4_]^+^ at *m*/*z* = 778.4932 Da (calcd *m*/*z* = 778.4931 Da) (Figure S3 in the Supporting Information). Random P3HT-*ran*-P3TEGT and block P3HT-*block*-P3TEGT copolymers were synthesized by using Kumada–Tamao catalyst-transfer condensation polymerization (CTCP). However, P3HT-*block*-P3TEGT was prepared by the sequential addition of the activated 2-bromo-3-hexyl-5-iodothiophene and 2,5-dibromo-3-(2-(2-(2-methoxyethoxy)ethoxy)ethoxy)methylthiophene monomers to a nickel catalyst (Figure 3B) while for P3HT-*ran*-P3TEGT, the two monomers were added simultaneously (Figure 3C). The SEC profiles of P3HT-*block*-P3TEGT and P3HT-*ran*-P3TEGT are given in Figures S5 and S7 in the Supporting Information. The compositions of the two copolymers were determined by ^1^H NMR spectroscopy based on the characteristic peaks of P3HT (δ = 0.5–2.8 ppm) and P3TEGT (δ = 3.2–4.8 ppm) [62]. The weight fractions of P3HT were estimated to be 81% and 55% in P3HT-*block*-P3TEGT and P3HT-*ran*-P3TEGT (Figures S4 and S6 in the Supporting Information).

The critical micelle concentration (CMC) of the amphiphilic thiophene-based surfactants was measured by scattered light intensity (Figures S8–S10 in the Supporting Information). The results were visualized by plotting the scattered light intensity against concentration, revealing a sharp increase at the CMC. This method relies on the principle that larger particles scatter light more effectively than small molecules. Solutions of the synthesized surfactants were prepared at varying concentrations and the CMC values were determined using dynamic light scattering (DLS). The copolymers P3HT-*ran*-P3TEGT and P3HT-*block*-P3TEGT display significantly lower CMC values, approximately 0.05 mM and 0.002 mM, respectively, compared to 3HT-3TEGT (0.13 mM). This difference can be attributed to the larger number of hydrophobic thiophene units (around 15) in the copolymers, compared to only three units in 3HT-3TEGT. Between the random and block copolymers, the higher CMC observed for P3HT-*ran*-P3TEGT is due to the greater proportion of the hydrophilic P3TEGT fraction in comparison to P3HT-*block*-P3TEGT.

### 3.2. Biodegradability Potential of Surfactant Compounds

Surfactants could be resilient to biodegradation and they could impact on the WWTP efficiency by disrupting the microbial community involved in biological pollution treatment. In most cases, bacterial communities involved in pollutant biodegradation adapt to various chemical composition from wastewater [63,64], but in certain cases, microbial biodegradation activity was disrupted by the surfactants present in wastewaters [65]. The BOD_5_/COD ratio (Biodegradability Index) has been generally considered the “cut-off point” between biodegradable and non-biodegradable compounds, as potential pollutants.

The BOD_5_ and COD assays on surfactant compounds showed a medium biodegradability potential for the 3DT-3TEGT compound, but a high biodegradation potential for P3HT-*block*-P3TEGT and P3HT-*ran*-P3TEGT (Table 2). A BOD_5_/COD ratio higher than 0.5 is an indication of a compound with high biodegradable potential (Table 1).

The biodegradation potential of these surfactants and their impact on the environment were correlated with the microbial respiration rate in the presence of each surfactant compound. The microbial respiration rate indicates the biodegradability of the surfactants. A reduced respiration rate serves as an indicator of the potential toxic effects of chemical compounds on bacterial communities from the activated sludge.

**Figure 4:**
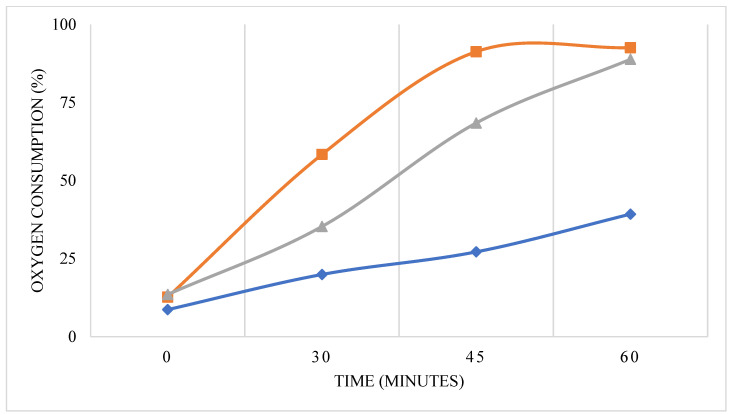
The effect of surfactants 3DT-3TEGT (blue line), P3HT-*block*-P3TEGT (orange line) and P3HT-*ran*-P3TEGT (grey line) on microbial oxygen uptake. All studies represent one of two independent experiments.

This method provides information on inhibitory or stimulatory effects after a short exposure time (usually from 30 min to 180 min or even longer) of the tested materials (surfactant compounds) on microorganisms/bacteria from the activated sludge. An activated sludge sample collected from a municipal WWTP was incubated for up to 1 h in the presence of 3DT-3TEGT, P3HT-*block*-P3TEGT, and P3HT-*ran*-P3TEGT. Overall, oxygen uptake was higher when the activated sludge was incubated in the presence of surfactants compared to the control group, which consisted of activated sludge incubated without surfactants (Figure 4).

The activated sludge incubated in the presence of 3DT-3TEGT, P3HT-*block*-P3TEGT, or P3HT-*ran*-P3TEGT increased the oxygen consumption compared to the control (activated sludge incubated without surfactants). The P3HT-*block*-P3TEGT had the highest oxygen consumption rate up to 45 min when less than 10% dissolved oxygen remained compared to control. P3HT-*ran*-P3TEGT induced an oxygen consumption at a lower rate than P3HT-*block*-P3TEGT up to 45 min, but reached the same oxygen level as P3HT-*block*-P3TEGT after 60 min. This could be explained by some chemical structures from P3HT-*ran*-P3TEGT that were initially more difficult to be biodegraded, but after a certain point, all chemical compounds were biodegraded at the same level as P3HT-*block*-P3TEGT (Figure 4). Overall, P3HT-*block*-P3TEGT and P3HT-*ran*-P3TEGT seemed to be easily biodegraded by the bacterial community from the activated sludge collected from a municipal WWTP.

Based on the oxygen consumption rate, 3DT-3TEGT was also biodegraded by the bacterial community, but at a lower rate compared to P3HT-*block*-P3TEGT and P3HT-*ran*-P3TEGT. The oxygen consumption rate induced by 3DT-3TEGT seemed to increase after 45 min, which could be explained by the biodegraded 3DT-3TEGT compound becoming more easily microbiologically processed.

**Figure 5:**
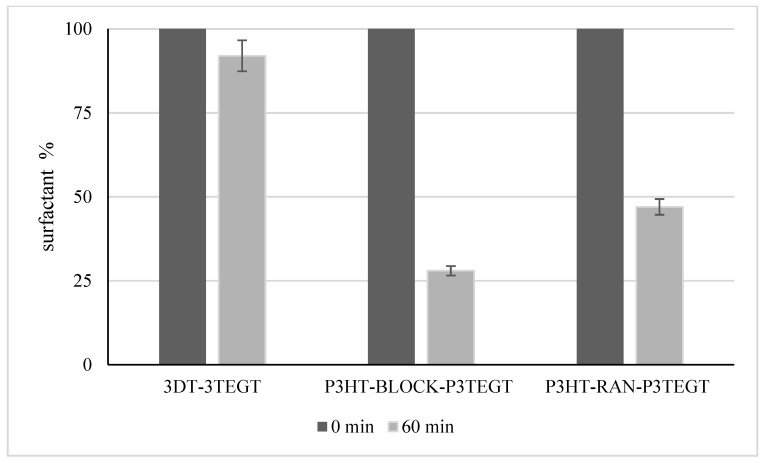
3DT-3TEGT, P3HT-*block*-P3TEGT, and P3HT-*ran*-P3TEGT surfactant quantification.

The oxygen consumption rate was correlated with the amount of surfactants, up to 60 min. The surfactant quantification after 1 h of incubation with activated sludge showed limited degradation for 3DT-3TEGT and more than 50% degradation for P3HT-*ran*-P3TEGT and up to 70% degradation for P3HT-*block*-P3TEGT (Figure 5).

P3HT-*block*-P3TEGT and P3HT-*ran*-P3TEGT compounds were properly correlated to the activated sludge respiration rate. The 3DT-3TEGT quantification for 60 min also matched the respiration rate (oxygen consumption) generated by the microbial communities from the activated sludge.

The structure of alcohol ethoxylates, such as RO(CH_2_CH_2_O)nH—a linear alcohol ethoxylate—have been often utilized in home applications and, therefore, they have been investigated in biodegradability tests [66].

The oxygen uptake was a measure of the ecotoxicological effect of surfactants on the bacterial metabolism’s potential to biodegrade 3DT-3TEGT, P3HT-*block*-P3TEGT, and P3HT-*ran*-P3TEGT. A higher oxygen uptake rate meant an active bacterial metabolism involved in surfactant biodegradation. Overall, these compounds did not exhibit any toxic effects on bacterial communities found in activated sludge from WWTPs. Furthermore, the surfactant compounds could be biodegraded during the standard treatment steps in the WWTP process, without the need to add specific bacterial strains to enhance their biodegradation.

### 3.3. Bacterial Growth Inhibition

**Figure 6:**
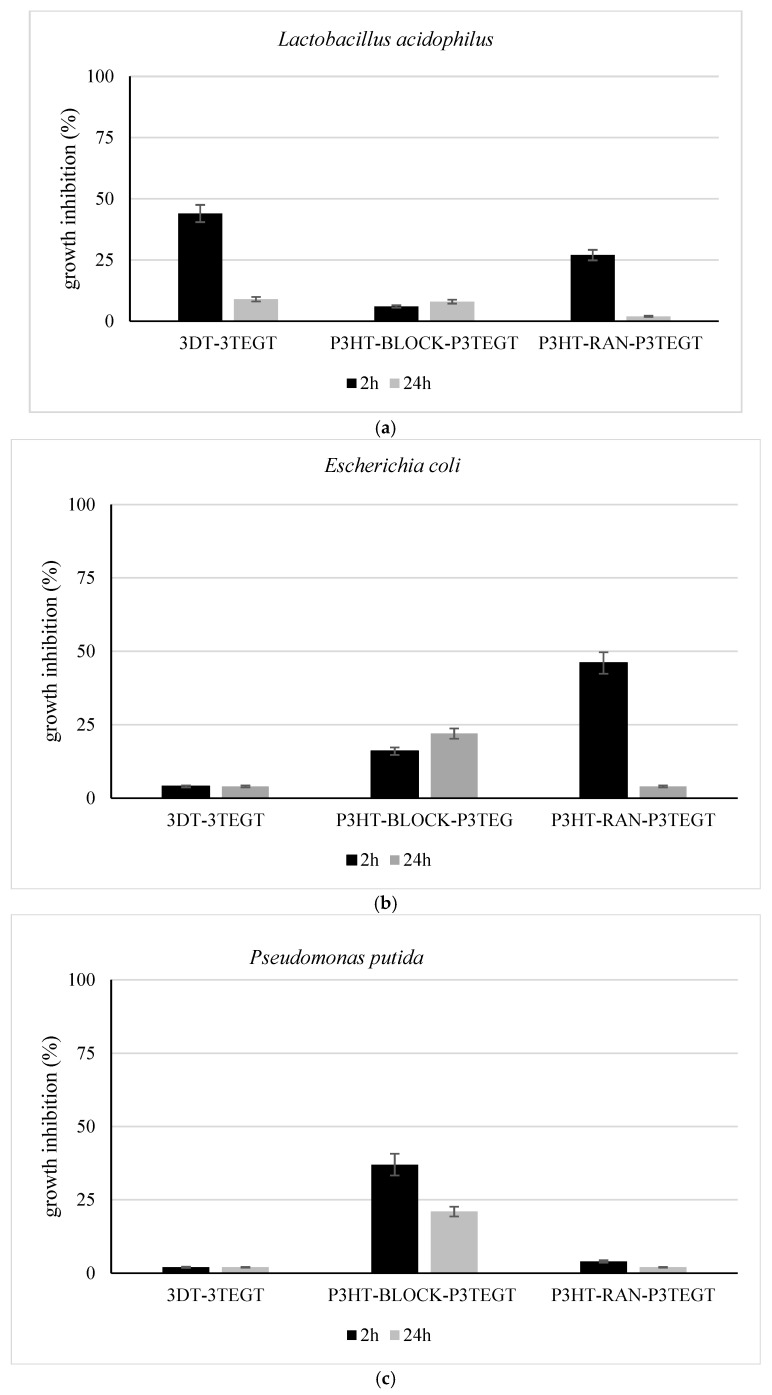
Bacterial growth in presence of 100 mg/L of surfactants. (**a**) *Lactobacillus acidophilus*; (**b**) *Escherichia coli*; (**c**) *Pseudomonas putida*. All studies represent one of three independent experiments.

The potential toxic effects of the newly designed and synthesized surfactant compounds (Figure 6) were analyzed by testing the bacterial growth inhibition on non-pathogenic bacterial models, including both Gram-positive and Gram-negative bacteria. Two Gram-negative bacterial strains (*E. coli* and *P. putida*) and one Gram-positive bacterial strain (*L. acidophilus*) were incubated in the presence of the three surfactants (3DT-3TEGT, P3HT-*block*-P3TEGT, and P3HT-*ran*-P3TEGT). The effect of surfactants on all three bacterial strains was examined by bacterial growth rate and viability. All three bacterial strains robustly grew in their specific growth medium, allowing for the ecotoxicity tests to be performed by incubating bacteria with various concentrations of the surfactant compounds (3DT-3TEGT, P3HT-*block*-P3TEGT, P3HT-*ran*-P3TEGT).

Results showed that at shorter incubation times, up to 2 h, a slight bacterial growth inhibition occurred, while over longer incubation times up to 24 h, the normal bacterial growth resumed, possibly due to bacterial adaptation mechanisms to the toxic effects of surfactants. The bacterial growth rate in these cases matched the control sample, where bacteria were incubated without 100 mg/L of 3DT-3TEGT, P3HT-*block*-P3TEGT, or P3HT-*ran*-P3TEGT, for extended incubation times (Figure 6). This effect was apparent across all bacterial strains, whether Gram-positive or Gram-negative, during incubation with 100 mg/L of surfactants. The specificity of the effects between the compounds and bacterial strains was more clearly observed at short incubation time (2 h).

The 3DT-3TEGT appeared to have a specific effect on the Gram-positive *L. acidophilus* strain, especially during a shorter incubation time (2 h), when bacteria growth inhibition reached up to 50%. At 24 h of incubation, the growth rate of *L. acidophilus* recovered, showing less than 10% inhibition compared to control samples.

The 3DT-3TEGT had an observable effect on *L. acidophilus*, but a limited one on the other two bacterial strains. However, it was reported a specific growth inhibition of Gram-positive *Staphylococcus aureus* incubated in presence of 180 ng/mL oligo (thiophene ethynylene) compound during 30 min incubation time, rather than at a longer incubation time (60 min) [67]. In contrast, at short and long incubation times P3HT-block-P3TEGT seemed to affect more the Gram-negative strains (*E. coli* and *P. putida*) compared to the Gram-positive strain. In addition, it exhibited a specific persistent toxic effect on *E. coli* during a longer incubation time (24 h) compared to *P. putida,* which recovered after 2 h of incubation. P3HT-*ran*-P3TEGT induced an insignificant growth rate inhibition to *P. putida*, but generated a similar growth inhibition pattern—a lower toxic effect for longer incubation times—for *E. coli* and *L. acidophilus* (Figure 6).

The toxicity effect of various surfactants on *P. putida* growth revealed that Gram negative bacteria showed lower sensitivity compared to marine microalgae *P. tricornutum,* [68]. It seemed that aggregation of surfactant molecules into micelles could cause oxygen diffusion limitations, which, in turn, negatively affects the Gram-negative bioluminescent bacterium *Photobacterium phosphoreum* [69]. Overall, the ecotoxicity tests on specific biological models revealed that 3DT-3TEGT, P3HT-*block*-P3TEGT, and P3HT-*ran*-P3TEGT did not significantly influence Gram-positive and Gram-negative bacterial strains over longer incubation times.

### 3.4. Life Cycle Assessment

LCA data collection was obtained from lab researchers directly involved in the synthesis of oligomeric and polymeric surfactants, as well as experts from the host institute. The collected data were subsequently used for life cycle inventory (LCI) and relevant data can be found in Tables S1 and S2 of the Supporting Information.

A contribution analysis was conducted to identify key contributors or stages with high environmental impacts, followed by recommendations for improvements in the synthesis process, based on LCA results.

**Figure 7:**
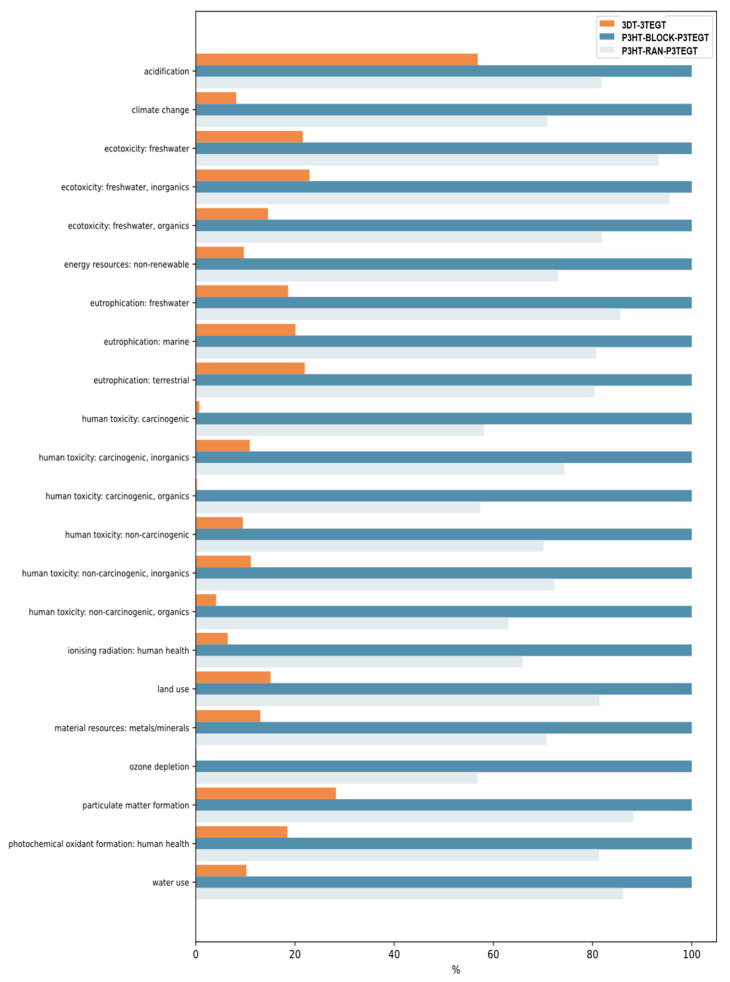
LCA impact analysis of 3DT-3TEGT, P3HT-BLOCK-P3TEGT, and P3HT-RAN-P3TEGT synthesis process on human and environmental health.

LCA results pointed out that 3DT-3TEGT exhibits a significantly lower overall environmental impact compared to P3HT-*block*-P3TEGT and P3HT-*ran*-P3TEGT (Figure 7). This disparity could be attributed to the use of organic solvents for synthesizing P3HT-*block*-P3TEGT and P3HT-*ran*-P3TEGT, as well as the overall efficiency of the synthesis process, the choice of catalysts, and the properties of the final products. For instance, P3HT-*block*-P3TEGT and P3HT-*ran*-P3TEGT require extensive use of organic solvents in their production (i.e., large amounts of chloroform and other organic solvents such as methanol, acetone, hexane, in the quenching and purification stage), which raises both energy demands and complexity in waste management, thereby increasing potential risks to ecosystems. In addition, P3HT-*ran*-P3TEGT performs better than P3HT-*block*-P3TEGT in each category due to reduced use of chemicals, such as M1, 1,3-bis(diphenylphosphino)propane]-nickel(II) dichloride, etc. The choice of palladium as a catalyst for 3DT-3TEGT enhances conversion rates, but it simultaneously increases the environmental burden associated with metal consumption.

In terms of the climate change impact, approximately 25–30% of the contribution from P3HT-*block*-P3TEGT and P3HT-*ran*-P3TEGT stems from the use of chloroform, illustrating the severe effects of using organic solvents. Furthermore, around 20% of the impact stems from high-voltage electricity and over 10% is associated with heat from natural gas. For 3DT-3TEGT, around 30% of the impact of electricity reflects the industrial process’s reliance on energy, emphasizing the importance of evaluating the environmental friendliness of electricity sources. Therefore, transitioning to cleaner energy sources in operations is recommended to mitigate environmental impacts further. Regarding the eutrophication impacts (freshwater) of all molecules, the 30% contribution from spoilage related to hard coal mining indicates that the environmental impacts associated with coal extraction and management are significant. The mining and disposal of hard coal can lead to contamination of soil and water, alongside detrimental effects on surrounding ecosystems. It was worth noting that for 3DT-3TEGT, the impact of using ethanol (from ethylene) contributed to around 40% of eutrophication.

In terms of ecotoxicity (freshwater), significant contributions (over 40%) came from water discharges linked to petroleum and natural gas extraction. This highlights a crucial need for improvements in water management and discharge strategies into aquatic systems.

Moreover, LCA studies showed that chloroform, a prominent carcinogenic source for humans, contributes to over 80% of impacts on humans when used in synthesis processes of P3HT-*block*-P3TEGT and P3HT-*ran*-P3TEGT.

This study emphasized the potential risks of using chloroform in the chemical synthesis of surfactants or other industrial applications, underscoring the need for optimization or finding alternatives surfactant synthesis protocols in accordance to green chemistry principles. Additionally, a solvent recovery strategy could enhance the sustainability of future synthesis processes by promoting a circular economy and enabling the reuse of materials.

## 4. Conclusions

Oligomeric and polymeric thiophene-based surfactants (P3HT-*block*-P3TEGT and P3HT-*ran*-P3TEGT) have significant potential for flexible electronic production, allowing the electron charge transport via their π-conjugated thiophene backbones. These ecofriendly surfactants from electronic devices could decrease the e-waste burden on the environment. Oligomeric and polymeric thiophene-based surfactants exhibited high biodegradability potential, confirmed by BOD_5_/COD analysis and respirometry tests. Oligothiophene-based surfactants (P3HT derivatives) showed a higher biodegradation potential than terthiophene-based surfactants (3DT-3TEGT). High biodegradability suggested a low environmental persistence, minimizing risks of bioaccumulation of these ecofriendly compounds.

The ecotoxicological tests carried out on bacterial biological models showed no significant toxicity toward bacterial communities (Gram-positive or Gram-negative) even under prolonged incubation. However, the hydrophobic dodecyl and hydrophilic ethylene-glycol groups from the terthiophene structure selectively slightly affected Gram-positive bacteria (lacking an outer membrane), unlike Gram-negative strains.

While the surfactants themselves did not have significant toxicity, the LCA analyses pinpointed some production steps that could be evolved to reduce the lifecycle impacts. The synthesis of P3HT-*ran*-P3TEGT and P3HT-*block*-P3TEGT had a higher footprint than 3DT-3TEGT due to the extensive use of chloroform, methanol, and hexane. In addition, chloroform emissions could contribute to the climate change trend. Climate change could also be boosted by cold-derived electricity, needed during the chemical syntheses of the surfactants. Coal mining and climate change could together impact on the environment (eutrophication and pollution) and human health.

Overall, this study underscores the potential of thiophene-based surfactants as sustainable alternatives for application in biosensors or organic electronics, but also emphasized the need for process improvements to align with green chemistry and circular economy goals. The LCA-identified hotspots could advance the synthesis of eco-friendly surfactants without compromising performance.

## Figures and Tables

**Table 1:** The Biodegradability Index.

BOD/COD Ratio	
<0.2	Non-biodegradable
0.2–0.5	Medium biodegradable
>0.5	Highly biodegradable

**Table 2:** COD and BOD_5_ values of 3DT-3TEGT, P3HT-*block*-P3TEGT, and P3HT-*ran*-P3TEGT.

Compounds/Parameters	COD	BOD_5_	BOD_5_/COD
	mg O_2_/L	mg O_2_/L	
3DT-3TEGT	2049	717	0.35
P3HT-*block*-P3TEGT	2091	1505	0.72
P3HT-*ran*-P3TEGT	2003	1121	0.56

## Data Availability

The original contributions presented in this study are included in the article/supplementary material. Further inquiries can be directed to the corresponding author.

## Supplementary Materials

The following supporting information can be downloaded at: https://www.mdpi.com/article/10.3390/ma18122701/s1, Figure S1: ^1^H NMR spectrum of 3HT-3TEGT (CDCl_3_, 400 MHz) at 298K.; Figure S2: ^13^ C{^1^H} NMR spectrum of 3HT-3TEGT (CDCl_3_, 126 MHz) at 298K; Figure S3: High-Resolution ESI-TOF (positive mode) mass spectrum of 3HT-3TEGT; Figure S4: ^1^H NMR spectrum of P3HT-*block*-P3TEGT (CDCl_3_, 400 MHz) at 298K; Figure S5: SEC profiles obtained during the synthesis of the P3HT-*block*-P3TEGT copolymer in THF; Figure S6: ^1^H NMR spectrum of P3HT-*ran*-P3TEGT (CDCl_3_, 400 MHz) at 298K; Figure S7: SEC profile of the P3HT-*ran*-P3TEGT copolymer in THF; Figure S8: Plot of the intensity of scattered light (in kilo counts per second) obtained for various concentrations of 3HT-3TEGT prepared in deionized water. The intersection of the two lines in the intensity data corresponds to the critical micelle concentration; Figure S9: Plot of the intensity of scattered light (in kilo counts per second) obtained for various concentrations of P3HT-*block*-P3TEGT prepared in deionized water; Figure S10: Plot of the intensity of scattered light (in kilo counts per second) obtained for various concentrations of P3HT-*ran*-P3TEGT prepared in deionized water; Table S1: Life cycle inventory (LCI) for 3DT-3TEGT; Table S2: Life cycle inventory (LCI) for P3HT-*block*-P3TEGT and P3HT-*ran*-P3TEGT.
