# Supplementary material for: Green Design and Life Cycle Assessment of Novel Thiophene-Based Surfactants to Balance Their Synthesis Performance and Environmental Impact
Source: Materials (Basel). 2025 Jun 8;18(12):2701. doi: 10.3390/ma18122701 (PMC12194560; doi:10.3390/ma18122701)
Supplement: Supplementary file 1 [file materials-18-02701-s001.zip › materials-3615336-supplementary.pdf]

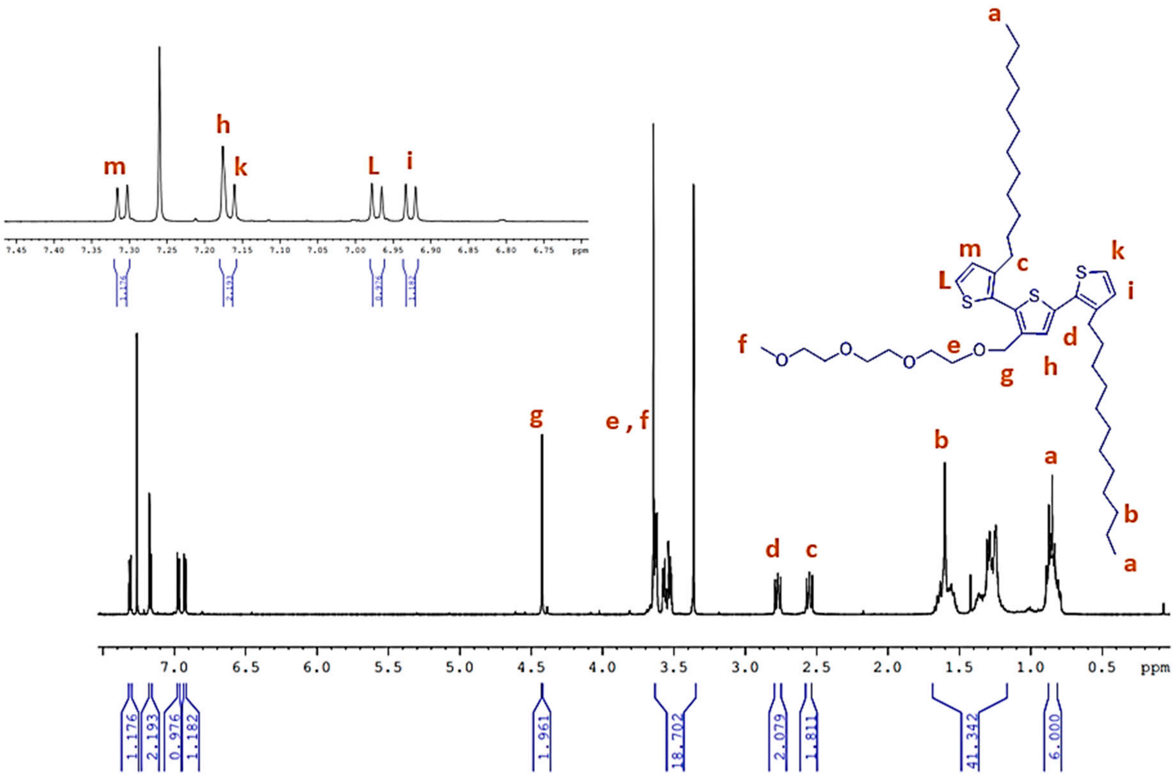

Figure S1. ^{1}H NMR spectrum of **3HT-3TEGT** ($CDCl_3$, 400 MHz) at 298K.

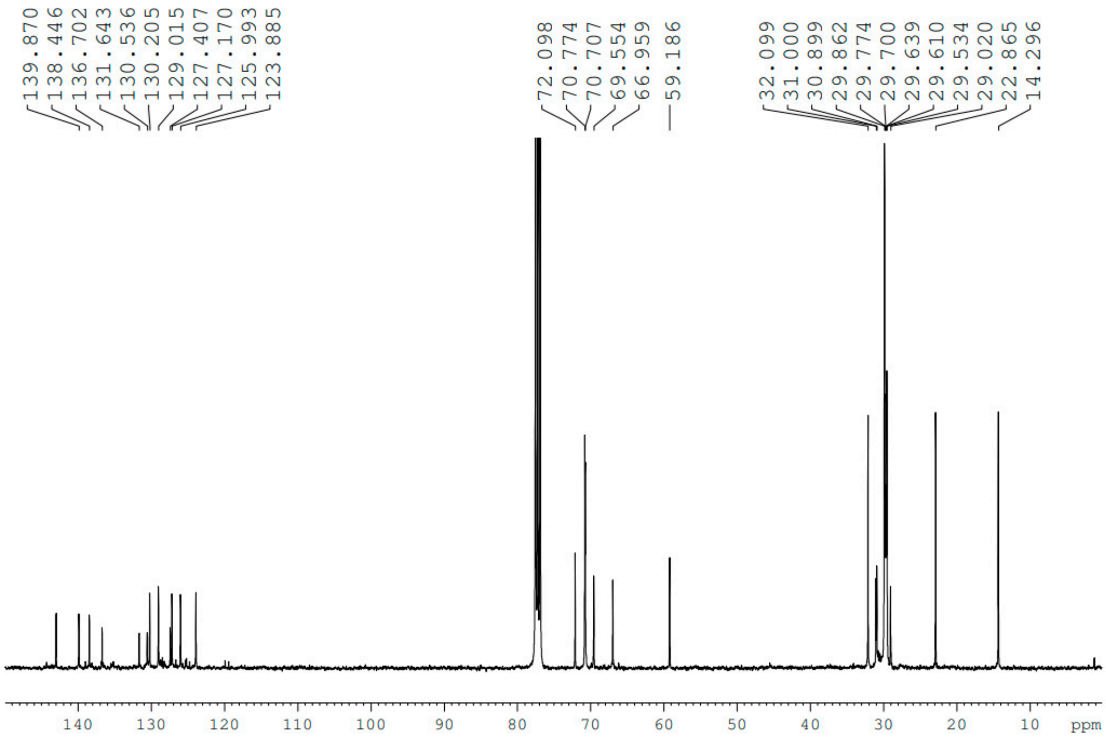

Figure S2.[13] C{^{1}H} NMR spectrum of **3HT-3TEGT** ($CDCl_3$, 126 MHz) at 298K.

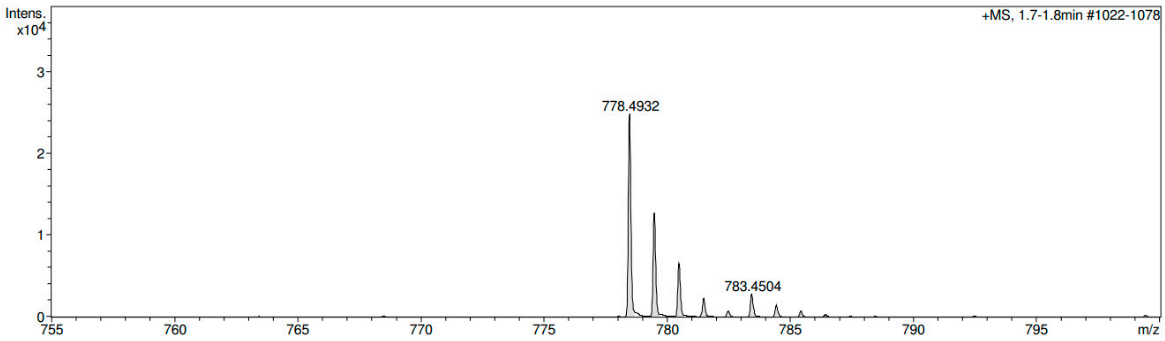

Meas. m/z	#	Ion Formula	m/z	err [ppm]	mSigma	# Sigma	Score	rdb	e⁻ Conf	N-Rule
778.4932	1	C44H76NO4S3	778.4931	-0.2	44.3	1	100.00	7.5	even	ok
	2	C40H72N7O2S3	778.4904	-3.6	45.5	2	16.07	8.5	even	ok
	3	C45H72N5S3	778.4944	1.5	45.7	3	56.13	12.5	even	ok
783.4504	1	C44H72NaO4S3	783.4485	2.4	10.8	1	48.77	8.5	even	ok
	2	C45H68N4NaS3	783.4498	0.7	16.0	2	100.00	13.5	even	ok

Figure S3. High-Resolution ESI-TOF (positive mode) mass spectrum of **3HT-3TEGT**.

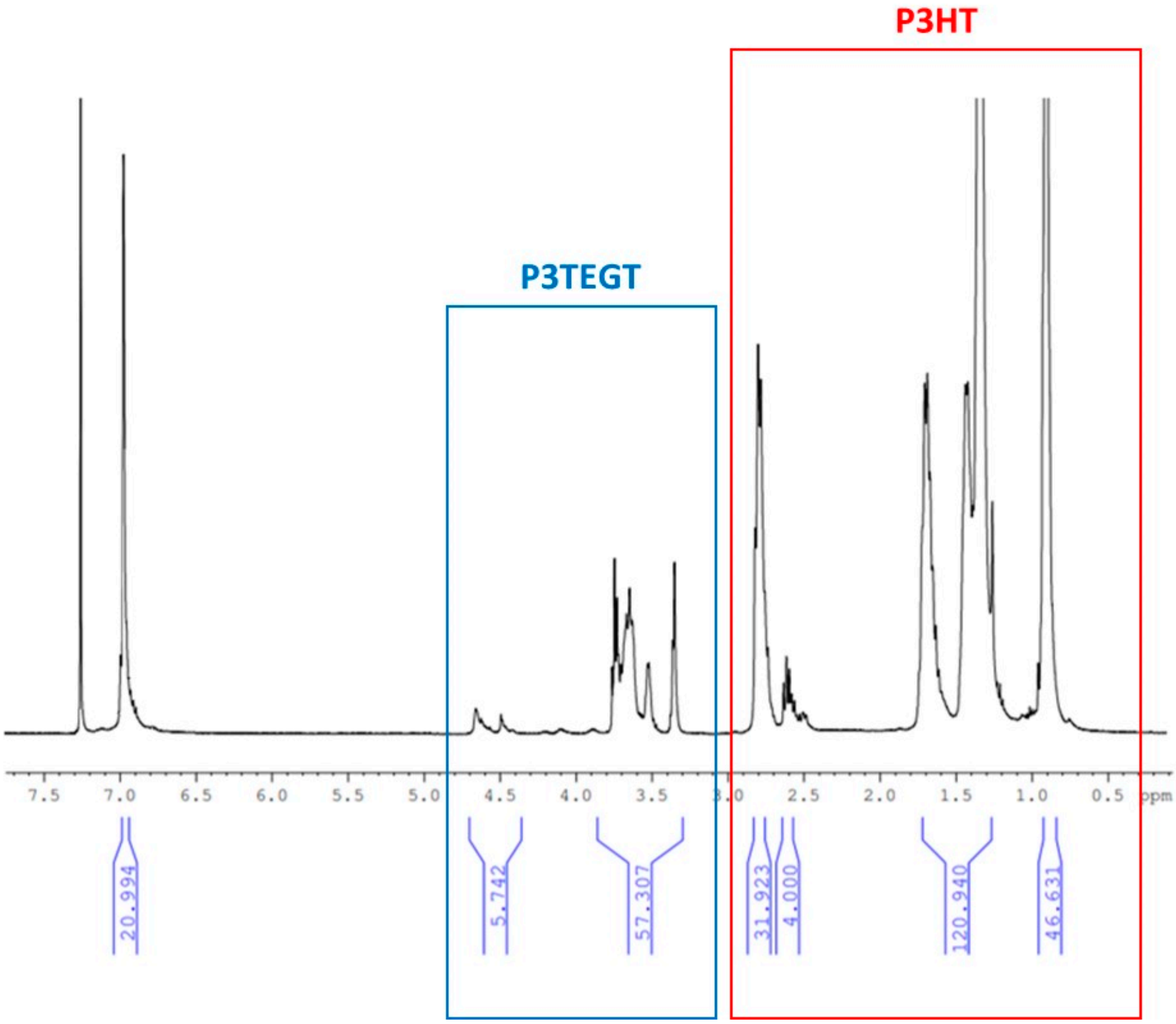

Figure S4. ^{1}H NMR spectrum of **P3HT-*block*-P3TEGT** ($CDCl_3$, 400 MHz) at 298K.

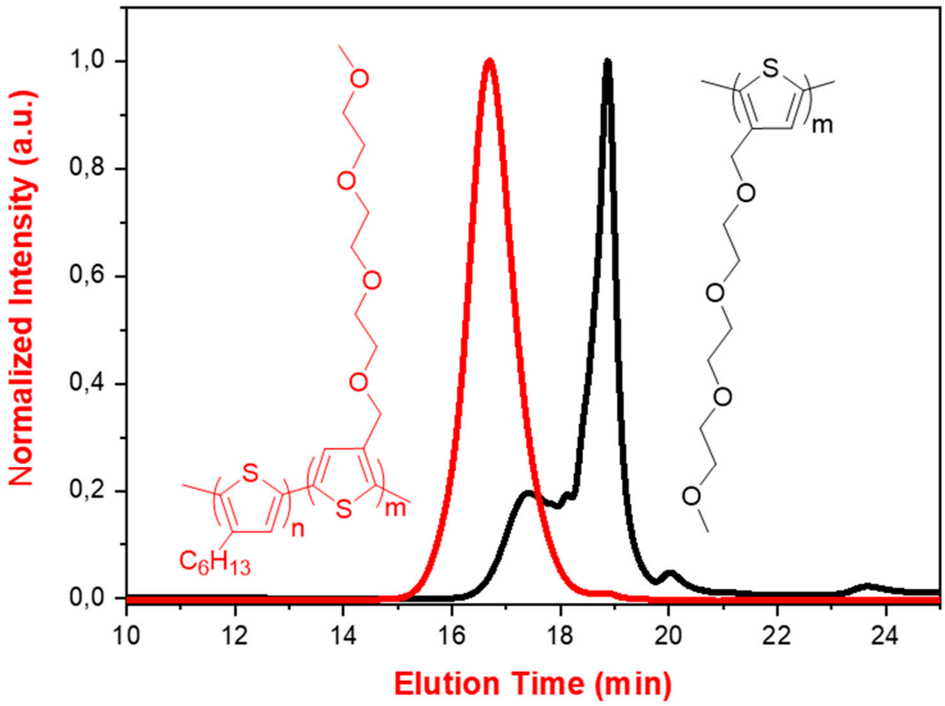

Figure S5. SEC profiles obtained during the synthesis of the **P3HT-*block*-P3TEGT** copolymer in THF.

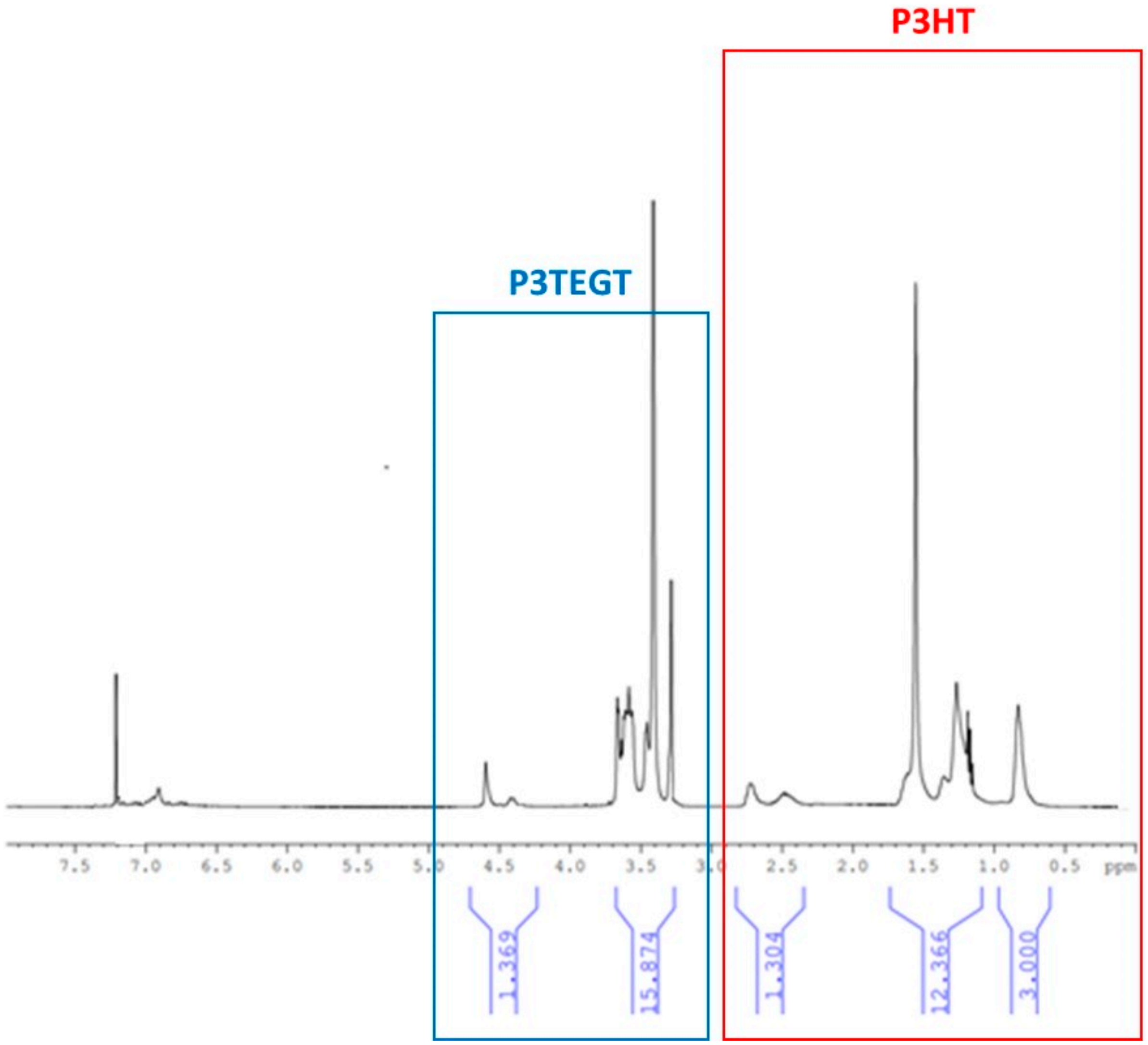

Figure S6. ^{1}H NMR spectrum of **P3HT-*ran*-P3TEGT** ($CDCl_3$, 400 MHz) at 298k.

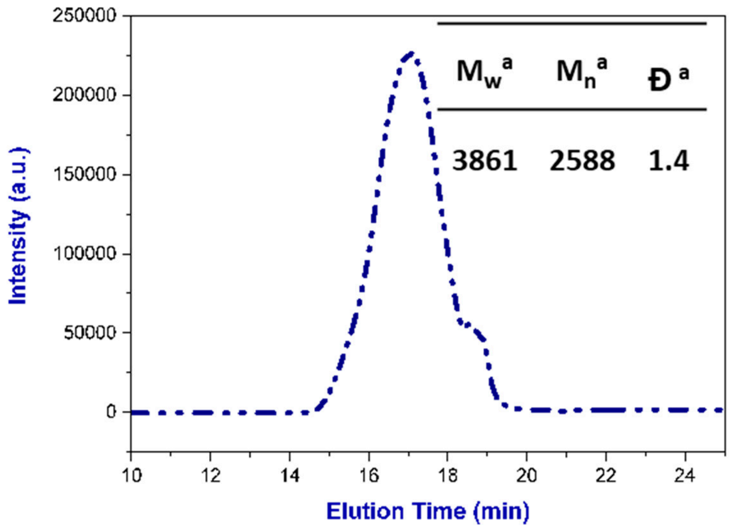

Figure S7. SEC profile of the **P3HT-*ran*-P3TEGT** copolymer in THF.

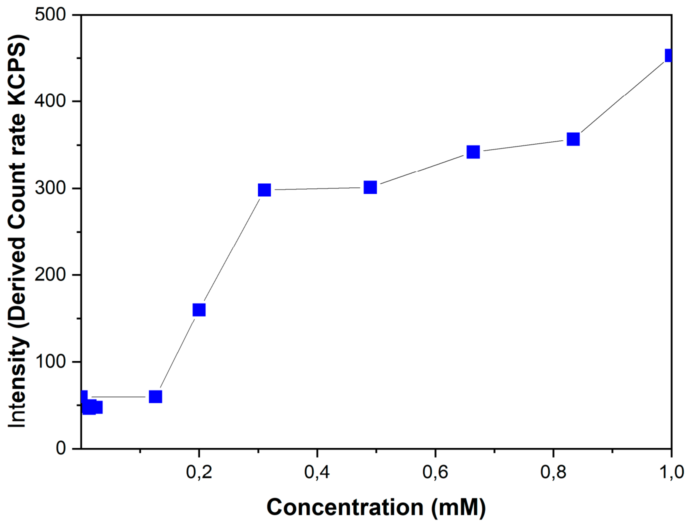

Figure S8. Plot of the intensity of scattered light (in kilo counts per second) obtained for various concentrations of **3HT-3TEGT** prepared in deionized water. The intersection of the two lines in the intensity data corresponds to the critical micelle concentration.

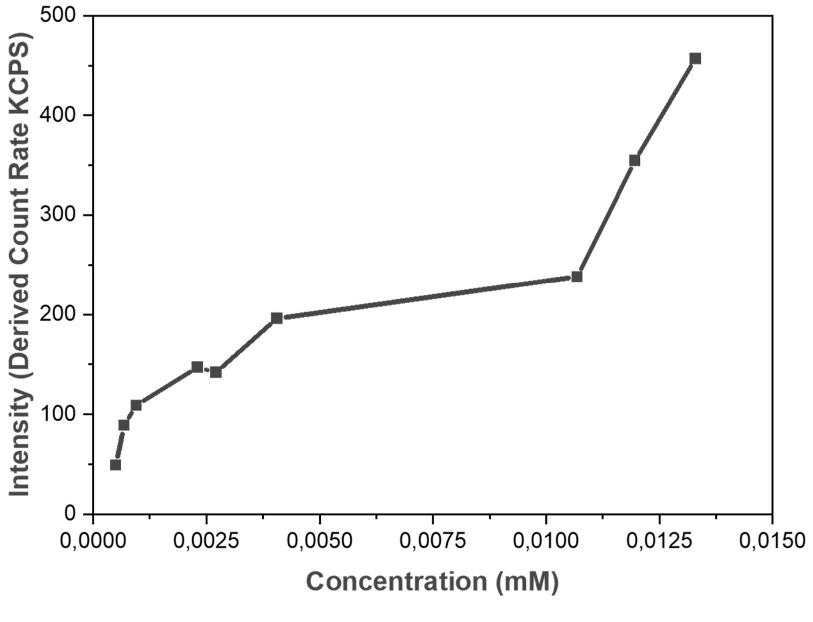

Figure S9. Plot of the intensity of scattered light (in kilo counts per second) obtained for various concentrations of **P3HT-*block*-P3TEGT** prepared in deionized water.

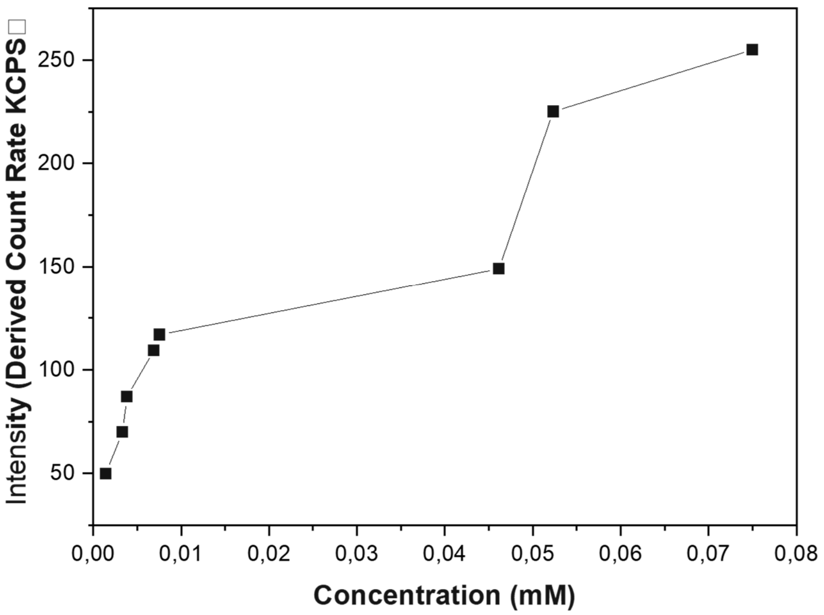

Figure S10. Plot of the intensity of scattered light (in kilo counts per second) obtained for various concentrations of **P3HT-*ran*-P3TEGT** prepared in deionized water.

Table S1. Life cycle inventory (LCI) for 3DT-3TEGT

Synthesis step	Unit operation	Chemicals	Unit	Amount	Time [min]	Condition	Energy [kWh]	Device	Yield	Other
1. Synthesis of 2-bromo-3-dodecylthiophene (a) C16H27BrS										
1.1	Dissolution	Dodecyl thiophene	g	3				Beaker		
		Glacial acetic acid	mL	50						
1.2	Heating	Recrystallized N-Bromosuccinimide, NBS	g	2.4	30	45 °C		Heating Plate		
1.3	Extraction	H2O	mL	100						
		ether	mL	100						
1.4	Washing	NaOH	M	2		3 times				
		H2O	mL	100		3 times				
1.5	Drying	Anhydrous magnesium sulfate (MgSO4)	g	2						
1.6	Purification	Hexane:Ethyl acetate	L	1L:0.2L		160 °C, 0.1 mbar			0.66	
2. Synthesis of 2-(3-dodecylthiophen-2-yl)-4,4,5,5-tetramethyl-1,3,2-dioxaborolane (b) C22H39BO2S										
2.1	Reflux	2-bromo-3-Dodecyl Thiophene (compound a)	g	2				Beaker		
		Potassium Acetate	g	1.78						
		Bis(pinacolato)diboron	g	2.3						
		Palladium Catalyst (PdCl2(dPPf)2)	g	0.0018						
		1,4-dioxane	mL	60	1440	80 °C, N2		Heating plate		
2.2	Extraction	H2O	mL	30						
		Dichloromethane	mL	100						
2.3	Rotary evaporation	Anhydrous Na2SO4	g	3						
2.4	Purification	Hexane: Ethyl acetate	L	1L:1L				Column Chromatography	0.6	
3. Synthesis of 3-(2-(2-(2-methoxyethoxy)ethoxy)ethoxy)methylthiophene (c) C12H20O4S										
3.1	Stirring	3-methanol	g	3		benzaldehyde				
		1-(2-Bromoethoxy)-2-(2-methoxyethoxy)ethane	g	7.2						
		tertabutylamoniumbromide (TBAB)	mg	300						

		Water	g	4.5					
		DMSO	mL	5					
		Potassium hydroxide	g	3.4	1440				
3.2	Quenching	Water	mL	100 (Total)					
	Extraction	Ether	mL	5mL					
3.3	Washing	Water	mL	30 Ml					
3.4	Drying	anhydrous MgSO4	g	3					
3.5	Purification	Mixture of 7:3 Hexane:Ethyl acetate	L	1L:0.3L			Column chromatography	0.71	
4. 2,5-dibromo- 3-(2-(2-(2-methoxyethoxy)ethoxy)ethoxy)methylthiophene (d) C12H18Br2O4S -M1									
4.1	Stirring	Product (c)	g	2.3			Dry one-necked round bottom flask		
		Tetrahydrofuran (THF)	mL	100					
		N-Bromosuccinimide (NBS)	g	4.6	30	0 °C			
4.2	Reaction				60	0 °C	Left in RT overnight		
4.3	Filtration and washing	Hexane	mL	10 mL					
4.4	Drying	Magnesium sulfate	g	4					
4.5	Filtration and evaporation								
4.6	Purification	Hexane:Ethyl acetate mixture 8:2	L	1L:0.2L			Column chromatography	0.8	
5. General procedure for 3DT-3TEGT									
5.1	Stirring	Hydrophobic thiophene (b)	g	0.4					
		Hydrophilic thiophene (d)	g	0.22					
		THF	mL	12					
		Sodium carbonate solution	mL	4	10	RT			2 M
5.2	Reflux	Palladium catalyst	mg	5	1440	75 °C			
5.3	Quenching	Water	mL	50					
5.4	Extraction	Dichloromethane	mL	50					
5.5	Drying	Anhydrous MgSO4	g	3					
5.6	Rotary evaporation								

5.7 Purification	Hexane:Ethyl acetate	L	1L:0.2L	Column chromatography	0.5

Table S2. Life cycle inventory (LCI) for P3HT-block-P3TEGT and P3HT-ran-P3TEGT

Synthesis step	Unit operation	Chemicals	Unit	Amount	Time [min]	Condition	Energy [kWh]	Device	Yield	Other
3. Synthesis of 3-(2-(2-(2-methoxyethoxy)ethoxy)ethoxy)methylthiophene (c) C12H20O4S										
3.1	Stirring	3-methanol	g	3		benzaldehyde				
		1-(2-Bromoethoxy)-2-(2-methoxyethoxy)ethane	g	7.2						
		tertabutylamoniumbromide (TBAB)	mg	300						
		Water	g	4.5						
		DMSO	mL	5						
		Potassium hydroxide	g	3.4	1440					
3.2	Quenching	Water	mL	100 (Total)						
	Extraction	Ether	mL	5mL						
3.3	Washing	Water	mL	30 Ml						
3.4	Drying	anhydrous MgSO4	g	3						
3.5	Purification	Mixture of 7:3 Hexane:Ethyl acetate	L	1L:0.3L				Column chromatography	0.71	
4. 2,5-dibromo- 3-(2-(2-(2-methoxyethoxy)ethoxy)ethoxy)methylthiophene (d) C12H18Br2O4S -M1										
4.1	Stirring	Product (c)	g	2.3				Dry one-necked round bottom flask		
		Tetrahydrofuran (THF)	mL	100						
		N-Bromosuccinimide (NBS)	g	4.6	30	0 ℃				
4.2	Reaction				60	0 ℃		Left in RT overnight		
4.3	Filtration and washing	Hexane	mL	10 mL						
4.4	Drying	Magnesium sulfate	g	4						
4.5	Filtration and evaporation									
4.6	Purification	Hexane:Ethyl acetate mixture 8:2	L	1L:0.2L				Column chromatography	0.8	

5. General procedure for P3HT-block-P3TEGT								
5.1 Drying	M3 2-bromo-3-hexyl-5-iodothiophene	g	0.558	10	50 ℃			
	M1 (chemical d)	g	0.21	10	50 ℃			
5.2 Mixing	THF	g	0.89	15		0		
	Isopropylmagnesium chloride (iPrMgCl)	g	0.22	60		0		2 M
5.2 Mixing	$NiCl_2$(dppp) (C27H26Cl2NiP2), 1,3-bis(diphenylphosphino)propane]-nickel(II) dichloride	mg	18	60	RT			
5.3 Quenching	Methanol	mL	200	720	RT			
5.4 Purification	Methanol, Acetone, Hexane, Chloroform	mL	600, 300,300,300	5760	RT		0.54	
6. General procedure for P3HT-ran-P3TEGT								
5.1 Drying	M3	g	0.58	10	50 ℃			
	M1	g	0.62	10	50 ℃			
5.2 Mixing	THF	g	0.684	15		0		
	Isopropylmagnesium chloride	g	0.176	60		0		2 M
5.2 Mixing	$NiCl_2$(dppp) (C27H26Cl2NiP2)	mg	48	60	RT			
5.3 Quenching	Methanol	mL	200	720	RT			
5.4 Purification	Methanol, Acetone, Hexane, Chloroform	mL	600, 300,300,300	5760	RT		0.52	
